# Post Bellum Problems

**Published:** 1918-09

**Authors:** 


### Post Bellum Problems.

One of the biggest problems that the profession will have to meet, after the war, will be the professional spirit of the returning surgeons. Something like a third of the total active practitioners then in civil life, will come back to pick up their old practices or develop new ones, even if the military surgeons are not increased beyond the limits of those already commissioned and the number recently called. This is a misstatement according to medical directories but we believe it to be true so far as the actually practicing profession is concerned. This is a big problem by itself, as already discussed in the previous issue but it is not nearly so big as that depending on the spirit of the men returning.

These men will have been accustomed to an entirely different kind of discipline from that prevailing in peace, to an entirely different deference to prestige. Many who could not have gained prominence in civil practice, possessed attributes which have led to rapid rise in the army, implying such control of a medical or surgical service as is gained very slowly in civil life and only by a very few. Those who have not been so fortunate will, never-the-less feel disposed to assume the same attitude on their discharge. In spite of all that has been said regarding the relative unimportance of professional work in the limited sense in the army, the great majority will have had practices at least equal to what men claim but seldom realize in civil life. Not only will these men have had a large and wide experience, extending into various specialties and without reference to war surgery itself, to conditions seldom encountered in private practice by any one man except a specialist or one known to have a peculiar interest in a rare condition, but they will have become expert in various diagnostic and therapeutic technics usually left in civil life to a comparatively small number. Perhaps the greatest distinguishing feature will be the habituation to very early and long hours, to rapid though careful work, to a large clientele which comes without effort and which is subject to almost perfect control.

Thus far in the country's participation in the war, the influence of the withdrawal of large numbers of practitioners, is difficult to judge. From some places, the expected report of a prosperous and busy profession, is verified; from others comes the report of a dull winter and spring in spite of the removal of competition. It is impossible as yet, perhaps for the duration of the war, to harmonize conflicting reports and to judge as to actual conditions. It is self-evident, that unless other factors have acted to reduce professional work for those staying at home, there must be an average greater demand for professional services than for many years previously. With the meeting of two bodies of medical men, both accustomed to larger practices than prevailed before the war, there is bound to be sharp competition, which can scarcely be relieved even by the retention of many in the government service. We are apt to forget that the withdrawal of physicians from private into public service, even in the gradual development of state and local departments of health and similar enterprises, implies also the withdrawal of clientele, either directly or indirectly, by the reduction of sickness.

We have no intention of suggesting in detail the proper solution of the problem stated. What the profession, in and out of military service should grasp in advance, is the broad principle on which the solution must depend. To foresee the bald fact of competition is important in itself. Individually and as organized bodies, the profession must anticipate mutual competition in a broad and friendly spirit, both as to private practice and that in hospitals. The narrow, selfish scheming that has marred our past history must give place to a spirit of co-operation, fairness and generosity.

